# Analysis on effectiveness of subway epidemic prevention measures considering subway passenger flow

**DOI:** 10.3389/fpubh.2025.1627621

**Published:** 2025-08-01

**Authors:** Fang Zhou, Lanfen Lou, Fang Hou, Qiaoyun Ma

**Affiliations:** College of Information and Management Science, Henan Agricultural University, Zhengzhou, China

**Keywords:** SEIA, COVID-19, infectious disease transmission, Subway protection measures, asymptomatic infections

## Abstract

Since its discovery in 2019, the characteristics of COVID-19 have also changed, increasing pressure on subway authorities to control the spread of the disease. Most scientific contributions discussed the correlation between the movement of people in the subway and the spread of the virus, and provided ways to control the spread of the virus in the subway. However, in order to balance production and safety under the easing of the epidemic, it is necessary to establish a relatively common method to evaluate the effectiveness of subway epidemic prevention management measures. This paper investigates subway ridership data after the epidemic has eased, taking the subway of Z city as the case study. SEIA model, adapted from the SEIR (susceptible–exposed–infected–recovered) framework, was developed. The model divides infected people into symptomatic infections and asymptomatic infections, and simulates the movement of subway passengers to show the spread of COVID-19 in subways. In this study, the SEIA model was used to simulate the results of the three protection measures implemented by the subway management department of Z city, namely social distancing, reducing station stops and vaccination. The results showed that the measures of vaccination, while the measures of reducing station stops were the least effective.

## 1 Introduction

Since the global pandemic in 2020, the phenomenon of continuous mutation of COVID-19 has brought huge challenges to the security and development of countries around the world. Although there are many similarities between COVID-19 and the SARS coronavirus, which was discovered in 2002, COVID-19 was found to be more contagious (1, 2) and less lethal (2) in the 2020 study. The currently circulating Omicron variant is more contagious and less pathogenic than the original COVID-19 (3, 4), and it also has higher immunity against the vaccine (5). The highly contagious nature of COVID-19 is putting enormous pressure on public administrations. At the same time, asymptomatic cases of infection were detected in the early days of the pandemic. This population can only be detected by PCR, serologic testing, or lung scanning, and there are no obvious symptoms during infection (6). At present, asymptomatic infections account for a greater proportion of infected cases than symptomatic cases. Due to the large number of people and the difficulty of diagnosis, the number of asymptomatic infected people poses a great challenge to Chinese epidemic prevention work.

Research on the prevention and control of the COVID-19 epidemic mainly focuses on epidemiology, virology, medicine and other fields. Epidemiological research mainly uses mathematical modeling to predict and analyze epidemic epidemics, find and evaluate epidemic prevention measures; virology focuses on the isolation of virus structures, vaccine research and development; the medical aspect focuses on the study of the symptoms of COVID-19 (7) such as diagnosis and treatment. These research results have made important contributions to the formulation of epidemic prevention measures to curb the spread of the epidemic. However, for the government and relevant administrative departments, specific, precise, effective and fast-acting management measures are urgently needed to control the spread of the virus.

Controlling the source of the virus, cutting off the transmission route of the virus, and protecting the susceptible population are currently the main ways to prevent the spread of COVID-19. The effective measures to deal with it mainly include: daily epidemic prevention measures such as wearing masks, frequent disinfection, and ventilation; Isolation; imposing traffic control; when the epidemic is severe, it is necessary to take measures to close the city, such as the suspension of work in units and the online classes of students at home. But as the pandemic continues to evolve over the long term, there is an urgent need to strike a balance between social production and preventing the spread of the virus. With the gradual resumption of production and life, people must be vigilant against the possibility of the epidemic appearing at any time. Especially for commuting needs, the government encourages workers to choose walking, cycling, and self-driving commuting methods as much as possible to avoid crowd gatherings.

However, for big cities, the subway is an important part of daily travel. At the same time, the subway is also an important way for the spread of the virus. Local government authorities have adopted various daily epidemic prevention measures for urban public transport systems, which have played an important role in controlling the source of infection, cutting off the route of infection, and protecting vulnerable populations. For example, in the city of Wuhan, commuters need to scan a QR code to get on and off the bus. The scanning record of the QR code can be traced through the real-name system, which provides great convenience for the epidemic investigation. At the same time, Shanghai, Zhengzhou, Tianjin, Foshan and other cities have taken measures to suspend the operation of some lines. Although it is not difficult to find that different measures have sacrificed the efficiency of the normal use of the subway to varying degrees, the efficiency of some subways is worth sacrificing in order to ensure the safety of citizens. However, inefficient subway epidemic prevention measures not only affect the efficiency of the subway, but also fail to control the spread of the virus. Therefore, it is urgent to judge which subway epidemic prevention measures are truly effective. How to implement more accurate and effective prevention and control measures under normal epidemic prevention and control in the subway to ensure safe travel requires more in-depth research on the current transmission mechanism of COVID-19 in the subway.

In view of the aforementioned problem, this study simulated the spreading way of COVID-19 in the subway with a high concentration of people during the pandemic period by using a modified epidemiological model to evaluate the effectiveness of multiple subway epidemic prevention methods. Taking city Z as a case, the study investigated the subway data of city Z that resumed travel again after COVID-19 outbreak in 2022. The traditional SEIR model was changed to a model of the spread of COVID-19 inside the subway, and the spread of COVID-19 in the subway was simulated based on the surveyed subway data of city Z. The effectiveness of epidemic prevention measures was analyzed by simulating the impact of subway epidemic prevention measures such as social distancing, reducing station stops and vaccination on the changes in the number of people likely to be infected. It has contributed to the formulation of epidemic prevention measures in subway operations, and also provided a reference for other major public health emergency response measures.

## 2 Literature review

Through the investigation of subways, this study established a simulation model of the spread of COVID-19 among people who use subways. After determining the flow of people and the mode of virus transmission in the subway, the study simulated the implementation of subway epidemic prevention measures by changing the parameters of the model, and discussed the effectiveness of subway protection measures in controlling the spread of the virus. Such a prediction method can provide reasonable and effective preventive measures for the public health emergencies, so as to reduce the risk of decision-making.

In order to more accurately suppress the spread of the virus, some scholars have studied the relationship between the movement of people in public places and the spread of COVID-19 during the epidemic. Because COVID-19 is a virus that can be efficiently transmitted from person to person, scholars have paid attention to the changes in the population in various public places during the pandemic, such as university campuses (8), free parks (9), and public transportation (10). In the Madrid region of Spain, Tapiador L et al. found that people who frequently attend crowded public places, especially public transportation, have a high probability of overlapping with people infected with COVID-19 (11). Cartenì et al., by analyzing data published in Italy, found that the spread of the coronavirus was highly correlated with public transport trips (12). In the United States, the strong correlation between Metro data and the incidence of the disease has been highlighted in the literature (13). The above studies show that the movement of people in public places, especially public transport, is closely related to the presence of COVID-19 spreading. Therefore, a more detailed description of the relationship between the two can find targeted methods to control the spread of the virus. This study investigated the subway data of city Z in the field and analyzed the movement of subway riders during the COVID-19 pandemic. Through the analysis of one of the important components of urban public transportation, this paper provides suggestions for the suppression of urban virus transmission.

To control the spread of COVID-19 in the city through the management of the subway, there are investigates and studies on the subway epidemic prevention measures implemented. Some researchers have studied the conflict between the flow of passengers boarding and alighting in subway cars, and have looked for measures to minimize the conflict to reduce the spread of the virus (14). In order to reduce the spread of the virus caused by subway congestion, Rome has implemented measures such as limiting the number of people on the subway and gradually locking down and opening public events and facilities. Carrese et al. evaluated the measures in Rome and provided new recommendations (15). Some scholars have used mixed integer programming to provide peak-easing strategies for urban subway operations to achieve a balance between commuting needs and epidemic control (16). Many local governments are calling for social distancing when riding the subway, so scholars have studied the possible difficulties of social distancing in the subway and made new recommendations (17). Indoor air quality in metro stations is also an area of concern. Studies have suggested mitigation measures to improve indoor air quality in subways (18) to address the challenges of the coronavirus. Most of the studies have provided one or more effective metro measures or the improvement of existing measures, and consequently, it is more necessary to compare the effectiveness of these measures in controlling COVID-19 in the subway. On the basis of simulating the spread of the novel coronavirus among subway riders, this study simulated the effects of different subway epidemic prevention measures after implementation. Through the comparison of the epidemic prevention simulation effect of various measures, the subway management department can choose relatively effective epidemic prevention measures for implementation.

The epidemiological model SEIR has been widely used to analyze and predict the spread of the epidemic during the pandemic. As a classic model of infectious disease dynamics, many researchers have used the epidemiological model to predict the development of the pandemic in various regions after the outbreak of COVID-19 infection. For example, Efimov and Ushirobira used SEIR models and historical data to compare and predict the coronavirus outbreak in countries and regions such as France, Italy, Russia, New York State (US), and China (19). Ahmad et al. used the model to predict the direction of the epidemic in Pakistan over the next 200 days (20). At the same time, there are many articles that use the epidemiological model to discuss the effects of epidemic prevention measures. Because COVID-19 is currently predominantly transmitted from person to person, the SEIR model was changed to mimic close contact between people to discuss measures such as mask-wearing and reducing close contact (21). The model was also used to discuss the necessity and coverage for vaccination during the coronavirus outbreak in Brazil (22). Similarly, some researchers have divided infected patients into confirmed and unconfirmed infections on the SEIR model, and then divided confirmed infected patients into two states: hospital treatment and home isolation, in order to predict the development of the epidemic under different vaccination states (23). Rehman et al. also considered hospital treatment and case isolation based on the original SEIR model to further observe the evolution of the epidemic situation in Saudi Arabia (24). In addition to national and regional epidemic simulations, SEIR can also be simulated for specific public facilities such as subways. Some literatures have used the SEIR model as a theoretical basis to simulate the specific facilities and passengers’ trajectories in the subway in detail to find the risk factors in the subway (25). SEIR has also been used in a subway simulation in Xi’an, China, to discuss the impact of ventilation and disinfection, average commuter spacing, and commute duration on the spread of the virus in the subway (26). However, in view of the current development status of COVID-19, it is inevitable to establish a relatively general evaluation model of subway epidemic prevention and management measures. According to the development of COVID-19, the infected people were divided into symptomatic infections and asymptomatic infections in the model of this study. In order to simulate the flow of subway crowds, the model uses the subway data of city Z as a reference to increase the dynamic changes of passengers getting on and off the train. On this basis, by changing the corresponding parameters, we simulated the impact of the implementation of social distancing, reducing station stops and vaccination.

## 3 Background on the subway

As a rail transit built in the city and hauled by electricity, the subway has gradually become the dominant in urban rail transit because of its fast speed and large capacity. Due to the high value of urban land in large cities, the subway can save cost and space by building most of the urban rail transit underground (27). Because the tracks built underground do not overlap with other transportation systems, it is possible to avoid various buildings. At the same time, there will be no traffic jams during the subway operation, which can help passengers save a lot of commuting time. The capacity of the metro is 7–10 times more than that of busses. It travels at a high speed, generally 80 km/h, sometimes up to 120 km/h. As of December 31 (28), 53 cities in Chinese mainland have metro transportation services (28).

The Z city subway was used as a case for this subway epidemic prevention study. The six-car marshaling model was adopted by Z City Metro, which is the most widely used type of urban rail transit vehicle in China. Each carriage with a driver’s cab can generally accommodate 230 people, and the maximum passenger capacity can be 327. Each carriage without a driver’s cab can carry 250 passengers, and the maximum capacity is 352 people. The transport capacity can reach 25,000–50,000 passengers, and the average operating speed is 80 km/h, and the maximum speed is 120 km/h. However, the subway is less ventilated and breathable than ground traffic. Infectious diseases have made subway safety more serious challenges. In order to stop the spread of infectious diseases, it is important to study the transmission mechanisms of COVID-19, especially the way the disease spreads in the subway. By understanding the transmission mechanism and finding the key factors that affect the spread of COVID-19 in the subway can targeted protective measures be taken. The research method in this paper is to analyze the characteristics of subway passenger flow data and COVID-19 transmission, and establish a suitable model for simulation to reduce the spread of the disease.

Due to the selection of the subway in Z as the research object, the study collected the hourly passenger flow of Metro Line A and Line B in Z City in the week after the easing of epidemic as the research data. The hypothetical background of the study is that urban residents travel normally under the state of daily prevention and control, so the project collected subway data after the epidemic has eased as a reference for simulation. Based on such data characteristics, this paper uses model simulation to compare the effect of subway measures to control the spread of COVID-19.

After the project collected hourly passenger flow data, the trend of peak and off-peak passenger flow was clearly discovered. This difference in different scenarios may also lead to different results for different metro epidemic prevention measures, so they are discussed separately. Considering that some of the data involves the internal information of the subway operation in Z city, and the information cannot be directly disclosed, the article uses numeric and alphabetic names for the name of the running line and the date range to which the passenger flow belongs.

### 3.1 Hourly ridership

The hourly ridership of the subway can reflect the travel characteristics of passengers, which can help the operation department to make decisions and adopt the appropriate train marshaling plan. During the period of high passenger flow, the subway needs to increase the number of departures and reduce the interval between trains. Correspondingly, when the passenger flow is low, the subway reduces the frequency of departures, and still meets the travel needs of passengers and maintains the operation of the subway while saving costs. During the epidemic period and the period of normalized prevention and control, understanding the characteristics of passenger travel can help the subway and individuals to take effective protective measures while traveling.

**Figure 1:**
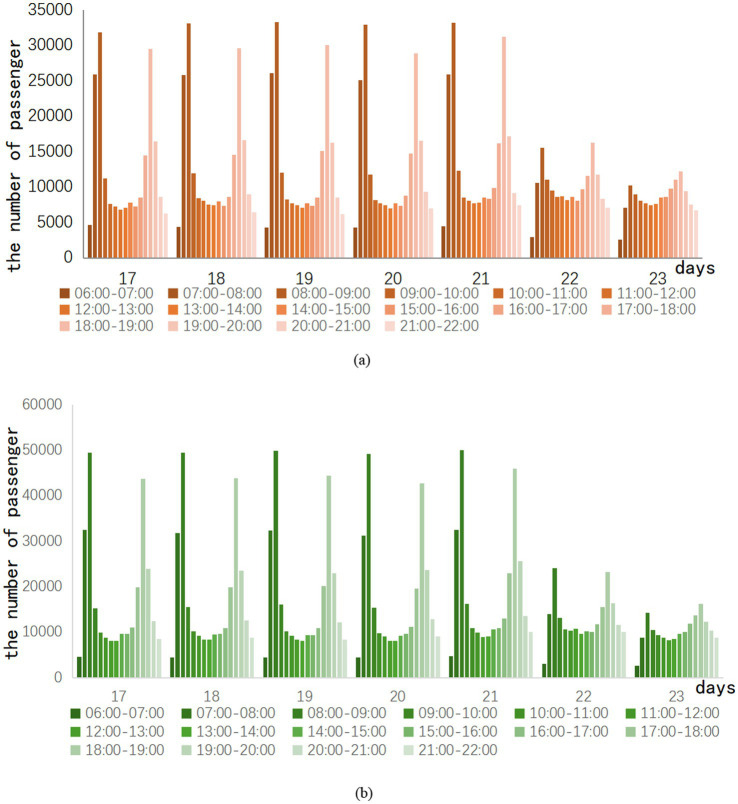
Hourly ridership in the week after the pandemic eased.

Therefore, the investigation took a pandemic outbreak in Z city as an example. After the outbreak of COVID-19, the week from day 17 to day 23 gradually came to an end. Passenger traffic on Routes A and B is also slowly returning to normal levels. The specific trend of ridership is shown in Figure 1.

In the week after the epidemic eased, the hourly passenger flow of Lines A and B had a clear trend of morning and evening peak changes every day. This indicates that the distribution of passenger traffic is concentrated in the morning and evening peaks. Hourly ridership peaks in the morning and evening when commuting demand resumes. In contrast, the flow variability in the morning peak is more apparent than in the evening peak. The 22nd and 23rd days are weekends, and the weekend traffic still has the morning and evening rush hours. However, the difference between peak and peak traffic on weekends is clearly not as obvious as on weekdays. For example, the 21st day is a Friday. There is a difference of 29,081 passengers between the highest and lowest passenger flow of the day on Line A, and 45,281 passengers between the highest and lowest passenger flow of the day on Line B. On the second day, there was only a difference of 13,406 passengers between the highest and lowest passenger flow of the day on Line A, and only 20,994 passengers between the highest and lowest passenger flow of the day on Line B.

Consequently, this study separates the peak data and the off-peak data in the hourly passenger flow to explore whether different subway epidemic prevention strategies will have different effects under the peak and off-peak conditions of subway passenger flow. By analyzing them separately, the study may give more accurate recommendations for subway epidemic prevention.

### 3.2 Ridership of peak

**Figure 2:**
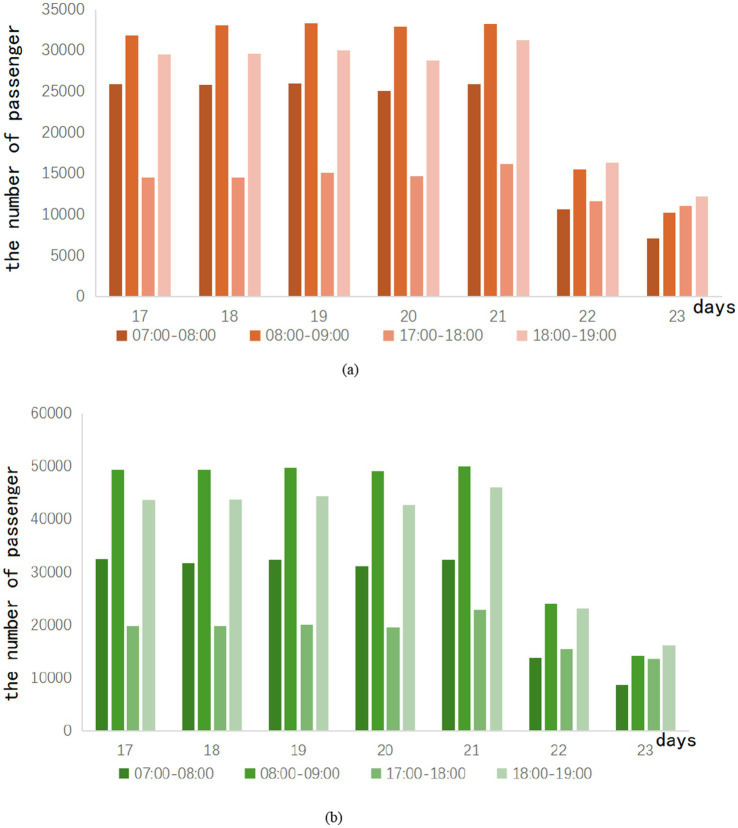
Ridership of peak in the week after the epidemic eased.

Peak hours refer to the time period when the passenger flow of the subway peaks during operating hours. Ridership during peak hours is greater than ridership in adjacent time periods. In the case of regular commuting, subway ridership tends to show a trend of morning peak and evening peak. During this period, subway operations often increase the transportation capacity of the subway by increasing the number of trains and shortening the departure interval. This avoids safety hazards and a bad passenger experience due to overcrowded trains. According to the surveyed hourly passenger flow, the peak period of city Z is judged to be 07:00–08:00, 08:00–09:00 in the morning, and 17:00–18:00 and 18:00–19:00 in the afternoon. The peak passenger flow of Lines A and B during the easing period of the epidemic can be shown in Figure 2.

In Figure 2, the weekend ridership of Lines A and B is about half of that on weekdays. During each weekday, the morning peak is always greater than the evening peak. This can be explained by the fact that some working people deliberately miss the evening rush hour when they leave work. This phenomenon may also be caused by the deliberate arrangement of off-duty time by the company in city Z. On weekends, most of the people who use the subway do not need to commute, so there are different situations on weekends.

### 3.3 Ridership of off-peak

**Figure 3:**
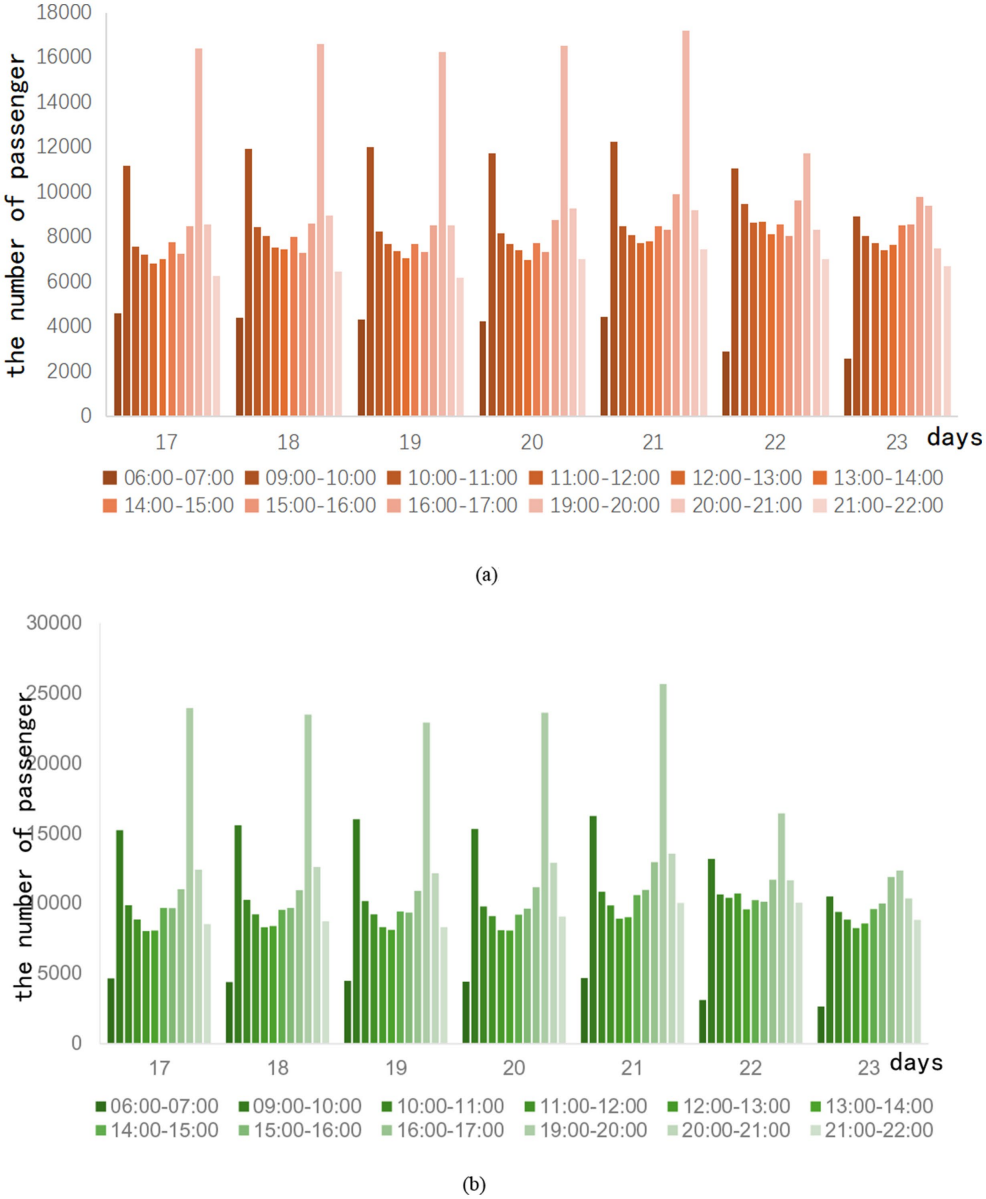
Off-peak ridership in the week after the epidemic eased.

During the running hours of the subway, the passenger flow is regarded as off-peak during the rest of the period. Ridership is usually average during this time period. When the epidemic situation in City Z enters a period of relaxation, the trend of subway passenger flow during peak hours is shown in Figure 3.

It is observed that the passenger flow trend of lines A and B is similar during the off-peak period. The 09:00–10:00 and 19:00–20:00 periods, respectively, peak during off-peak periods. Especially during the period from 19:00 to 20:00, the passenger flow of Line A can exceed 16,000 passengers, and the passenger flow of Line B is close to 25,000 passengers. After investigation, the reason for this phenomenon is that the subway operation has been calling for off-peak travel during the easing of the epidemic in Z City. As a result, some passengers choose to travel during these two times. Weekend traffic tends to be flatter, and although there is also a morning and evening peak, it is not marked compared to weekday traffic. The total number of trips on weekends is slightly higher than on weekdays. This shows that with the easing of the epidemic, the demand for non-essential travel is gradually recovering. After the epidemic, subway ridership is gradually recovering.

## 4 Methods

For the study, we established the SEIA model for the simulation of COVID-19 infection in the subway, which is based on the classical infectious disease model SEIR. The SEIR model involves four types of populations: susceptible population (S), exposed population (E), infected population (I), and removed population (R). The Equation 1 of the SEIR model is represented as follows:


(1) $$\left\{ \begin{array}{c} \frac{\mathrm{dS}}{\mathrm{dt}}=-\frac{\beta \times I\times S}{N}\\ \frac{\mathrm{dE}}{\mathrm{dt}}=\frac{\beta \times I\times S}{N}-\mathrm{\sigma E}\\ \frac{\mathrm{dI}}{\mathrm{dt}}=\mathrm{\sigma E}-\gamma \times I\\ \frac{\mathrm{dR}}{\mathrm{dt}}=\gamma \times I \end{array},\right.$$

N=S+E+I+R

**Figure 4:**
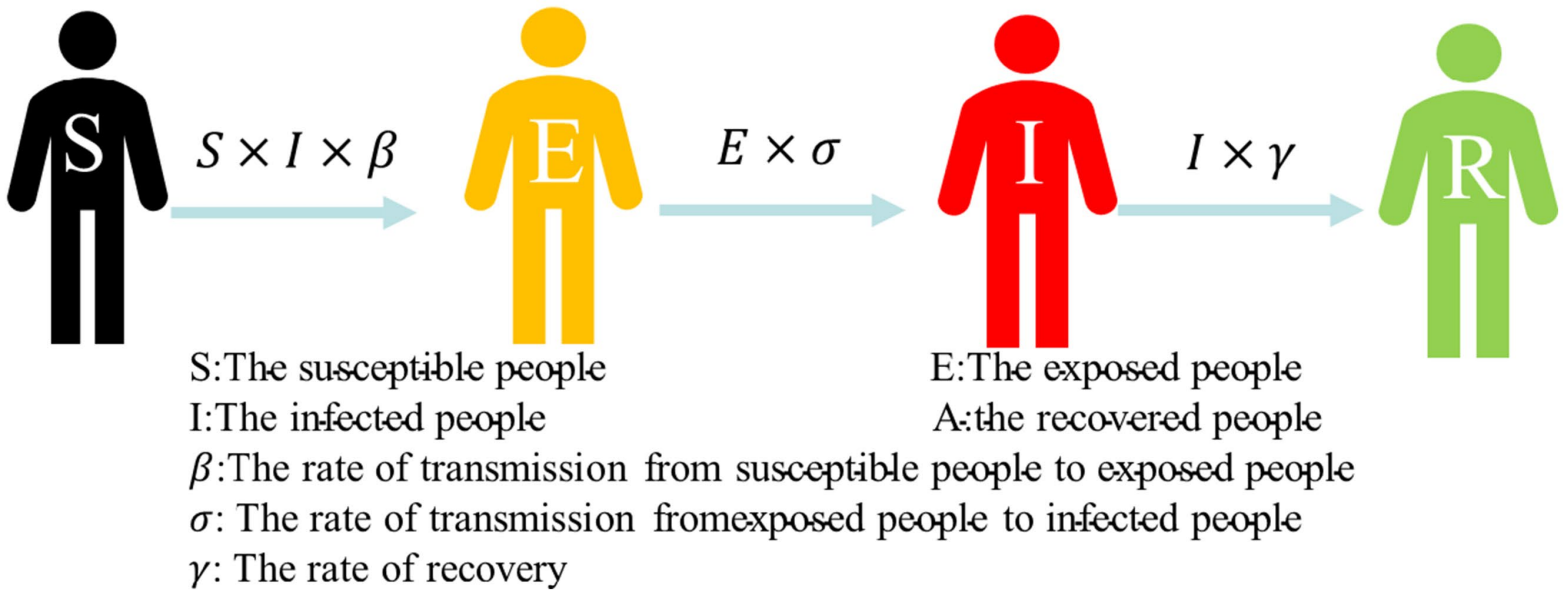
Susceptible–exposed–infected–recovered (SEIR) model.

Susceptible (S) individuals are transformed into exposed (E) individuals with a rate of β after contact with infected (I) individuals in the SEIR model. There is a rate of σ from exposed to infectious. Thus, infected exposed (E) individuals move from the exposed compartment to the infected compartment. Patients in the infected compartment are cured based on a rate of *γ* after treatment and become recovered (R) individuals. Consequently, they move from the infected warehouse and enter the recovered compartment. The transfer process is shown in Figure 4.

COVID-19 is a disease with latent periods, which susceptible groups need to be observed for an average of more than 7 days after contacting with an infected person to identify whether they have been infected with the disease (29). Therefore, the SEIR model provides a greater alignment with research requirements than infectious disease models such as SI and SIR, which adds the exposed (E) compartment. The basic assumptions of classical infectious disease models do not consider factors such as population migration and natural death, so they are suitable for the study of the short-term process of virus transmission in subway carriages. However, the SEIR model is versatile. In practical application during the pandemic, the simulation scenarios for metro travel need to be modified.

The improved SEIR model (SEIA) was constructed together with the symptomatic infections (I) and asymptomatic infections (A) as virus transmitters, and the model included four groups of people: Susceptible, Exposed, Infected, and Asymptomatic groups. In the case of the spread of the novel coronavirus in the subway, considering that passengers have limited time to take the subway, and there is a certain incubation period when the exposed population transforms into an infected population, the study can assume that the infected person in the subway will no longer transmit the virus to other susceptible groups in a single ride. Therefore, the transmission model in the subway only considers the process by which a susceptible population becomes an exposed population after coming into contact with an infected population. However, susceptible people do not must to be infected with the transmissible disease after contact with the infected person, so group of latent people is named as the exposed group. Since China has included asymptomatic infections in the daily new notification since April 2020, and asymptomatic infections are also transmissible, and the proportion of asymptomatic infections among infected people is gradually increasing, asymptomatic patients are classified as a separate group of people. The usual version of SEIR model consists of a system of differential equations. In order to grasp the characteristics of different subway stations running in different stations, a discrete compartment model is proposed in this study. Unlike continuous differential equation models, discrete models are more suitable for computer simulations and real-world data analysis (30). The simulation model for the spread of COVID-19 in the subway is shown by Equation 2.


$$S_{t+1}=S_{t}-\frac{r\times S_{t}(\delta_{1}I_{t}+\delta_{2}A_{t})}{N_{t}}+u_{t}\times \frac{S_{0}}{N_{0}}-g_{t}\times \frac{S_{t}}{N_{t}}$$

$$E_{t+1}=E_{t}+\frac{r\times S_{t}(\delta_{1}I_{t}+\delta_{2}A_{t})}{N_{t}}-g_{t}\times \frac{E_{t}}{N_{t}}$$

$$I_{t+1}=I_{t}+u_{t}\times \frac{I_{0}}{N_{0}}-g_{t}\times \frac{I_{t}}{N_{t}}$$

(2) $$A_{t+1}=A_{t}+u_{t}\times \frac{A_{0}}{N_{0}}-g_{t}\times \frac{A_{t}}{N_{t}}$$

$$N_{t}=S_{t}+I_{t}+A_{t}+E_{t}$$

In the formula, $S_{t}$ is the number of susceptible people at time t (a certain station) in the subway car. $E_{t}$ is the number of exposed people at time t in a subway car. $I_{t}$ denotes the number of symptomatic infected people at time t in a subway car. At is the number of asymptomatic infections at time t in a subway car. The variable r is the effective contact number of infected persons I and A who come into contact with susceptible persons S. $\delta_{1}$ denotes the rate that susceptible person S will come into contact with symptomatic infected person I and be infected. Similarly, $\delta_{2}$ is the rate that susceptible person S will come into contact with asymptomatic infected person A and be infected. According to the particularity of the subway environment, the variable $u_{t}$ is applied as the number of people boarding the subway at time t. At the same time, the variable $g_{t}$ is the number of people who get off the subway at time t. These two added variables can simulate the increase or decrease of the number of people in the carriage when the train stops, and then study the spread of the epidemic in the subway. $N_{t}$ is the sum of the number of people in the subway car at time t, which is composed of susceptible people, exposed people, symptomatic infected people and asymptomatic infected people at time t.

**Figure 5:**
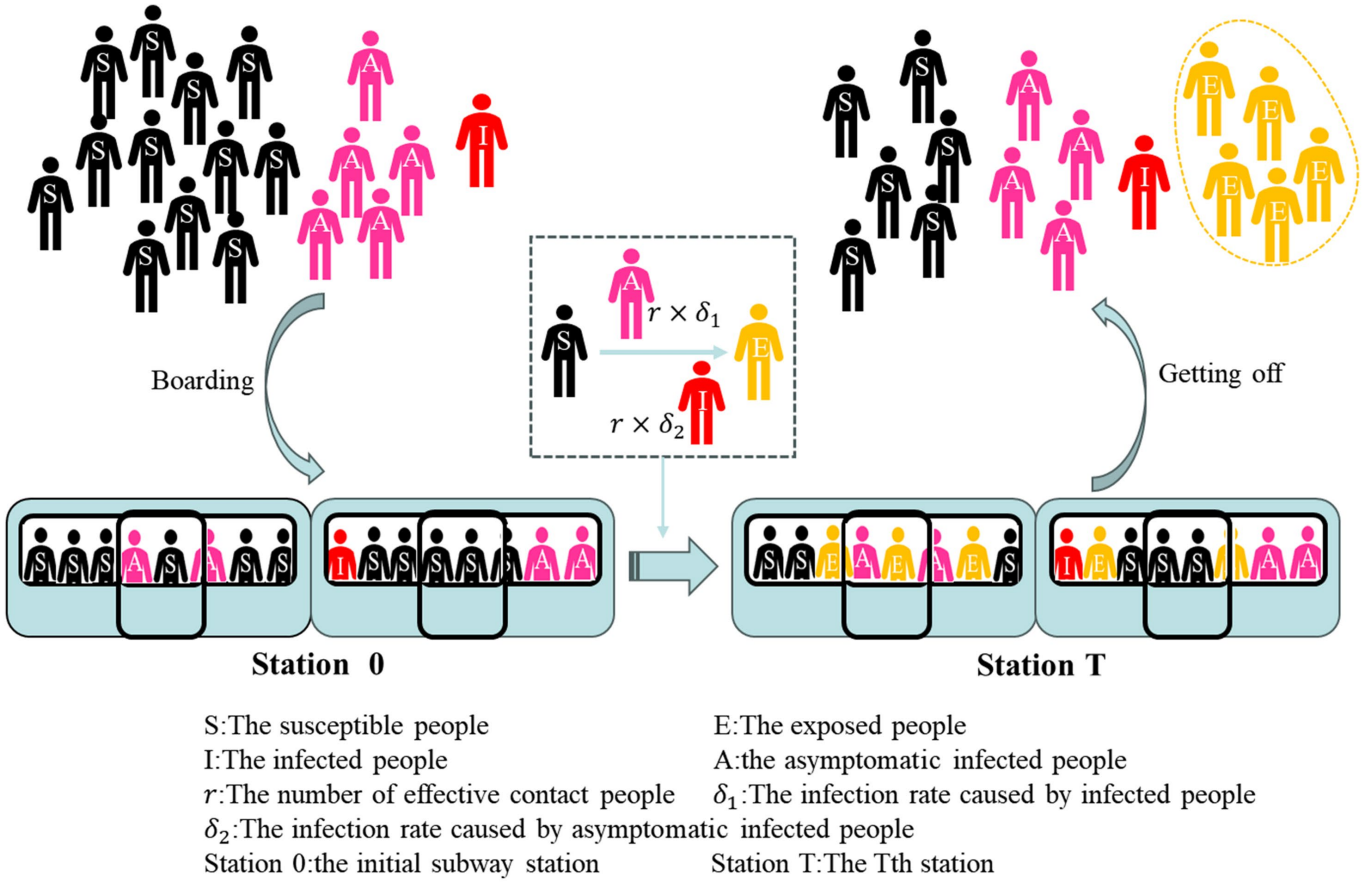
Flowchart of the proposed SEIA model for COVID-19.

The specific formula of the SEIA model expresses the change in the number of four groups of people in the subway car due to the spread of COVID-19 over time. This process of change can be seen in Figure 5. In studies of COVID-19, the median incubation time for close contact was 7 days (29). Similarly, it takes a period for an infected person to recover. Therefore, the main transformation that occurs during a short subway commute is that a susceptible person comes into contact with an infected person and becomes an exposed person. In order to effectively evaluate the risk of infection caused by subway commuting and simulate the implementation effect of subway epidemic prevention policy, simulation is designed to only observe that exposure group E is only generated during subway operation. Therefore, the exposed group in the crowd boarding the subway and in the crowd at the initial station of the subway is set to 0. $N_{t}$ at station 0 is quantitatively composed only of $S_{0}$ , $I_{0}$ , and $A_{0}$ . And when the subway starts running, the infection inside the subway car begins. When t is greater than 0, the exposed group in the total number of people begins to be greater than 0. And the crowd of people who go aboard the subway is also divided as susceptible people, infected people and asymptomatic infections. Under this design, only the cumulative number of exposed group E can be observed to assess the risk posed by metro operation during the COVID-19 pandemic. The people who get off the train are composed of four groups: susceptible people, infected people, asymptomatic infected people, and people who have been exposed after taking the subway.

During a subway operation, a propagation process was mainly carried out inside the subway car that susceptible group (S) is converted into exposed group (E) by contact with symptomatic infected group (I) or asymptomatic infected person A. The rate of susceptible people to exposed populations in the original SEIR model *β* is substituted by the rate of $r\times \delta_{1}$ and $r\times \delta_{2}$ in the SEIA model of subway simulation. This rate is made up of the product of the number of effective contact people and the infection rate of the infected group and the asymptomatic infected group, respectively. The purpose of splitting the parameters into two categories is to distinguish the infected group from the asymptomatic infected group, and at the same time provide conditions for the simulation of subway epidemic prevention countermeasures.

The data of the number of people getting on and off the train is based on the subway data of the field survey. The average number of people getting on and off at each station was calculated using multiple surveys of each station during the same period of the subway station in Z city. The average number of people boarding and alighting at each stop is $\lambda_{\mathrm{ut}}$ and $\lambda_{\mathrm{gt}}$ . The simulation uses MATLAB-R2022a to randomly generate the number of people getting on and off at each station following the Poisson distribution. The ratios of susceptible people, infected people, and asymptomatic infected people to the three population ratios at the initial station are assumed to be the same ratio in the simulation of the number of people boarding at each station. The study used the ratio of the average daily cumulative number of confirmed cases released by the Health Commission of Z City to the number of permanent residents in Z City in 2022 to approximate the ratio of the sum of the susceptible people to the infected people and the asymptomatic infected people in the subway boarding population. The ratio is calculated to be about 0.21%. In the daily cumulative number of confirmed cases released by the Health Commission of Z City during the same time period, the average ratio of infected people to asymptomatic infected people is 1:5. The simulation uses this ratio to approximate the proportion of infected people to asymptomatic infected people in the boarding population. And there are four types of people in the crowd that gets off the subway. Therefore, the proportion of various groups of people in the subway at the station are used to approximate the proportion of the number of people who get off the train at the station.

The established SEIA model was used to simulate the effects of different subway epidemic prevention measures under normal epidemic prevention, by changing the corresponding parameters. In the case of peak subway passenger flow and peak period, the transmission degree of different infection rates can be studied according to the different δ values of the infection rate of symptomatic infection and asymptomatic infection. If there are two groups of people with symptomatic infection and asymptomatic infection who have different degrees of impact on those who may be infected, then it is necessary to put forward corresponding protection recommendations for the group of people who cause greater transmission. The most important thing is to simulate three measures: social distancing, reduce stopped stations and vaccination to increase vaccine coverage. The model was used to explore the effect of these safeguards on the number of people group E. Simulate the implementation effect of the measure and provide reasonable suggestions for the subway, society and individuals.

## 5 Results

### 5.1 Simulation of the measure of social distancing

In less ventilated, confined spaces such as the subway, distancing is a common measure in controlling an epidemic. At the beginning of the disease, the government called for everyone to keep a distance of more than 1 m—to maintain a safe social distance. The railway authorities have adjusted the rail ticketing system to create spacing between people to reduce the risk of the spread of the epidemic. The Metro of city Z has also responded to the call of the state by calling on passengers who take the subway to keep a safe distance and sit at a distance as much as possible.

In response to the call of the subway operator, this study explores the impact of spacing measures on exposed people (E) by controlling the value of the infection rate of asymptomatic infected person $\delta_{2}$ . There are two reasons why $\delta_{2}$ is discussed to simulate interval sitting: on the one hand, studies have found that the infection rate of asymptomatic infected persons *β_2_* has a greater impact on the exposed people (E) (31). On the other hand, after symptomatic infection I develops symptoms, some people choose to avoid using less ventilated transportation or keep their distance from others when using the subway. In contrast, asymptomatic infected person A tends to move freely like a susceptible person because he has no associated symptoms, which may lead to inadvertent transmission of the epidemic.

**Figure 6:**
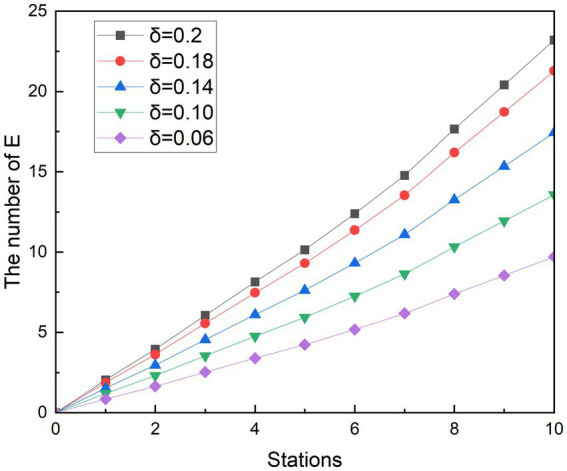
Simulation results of social distancing during peak period.

**Figure 7:**
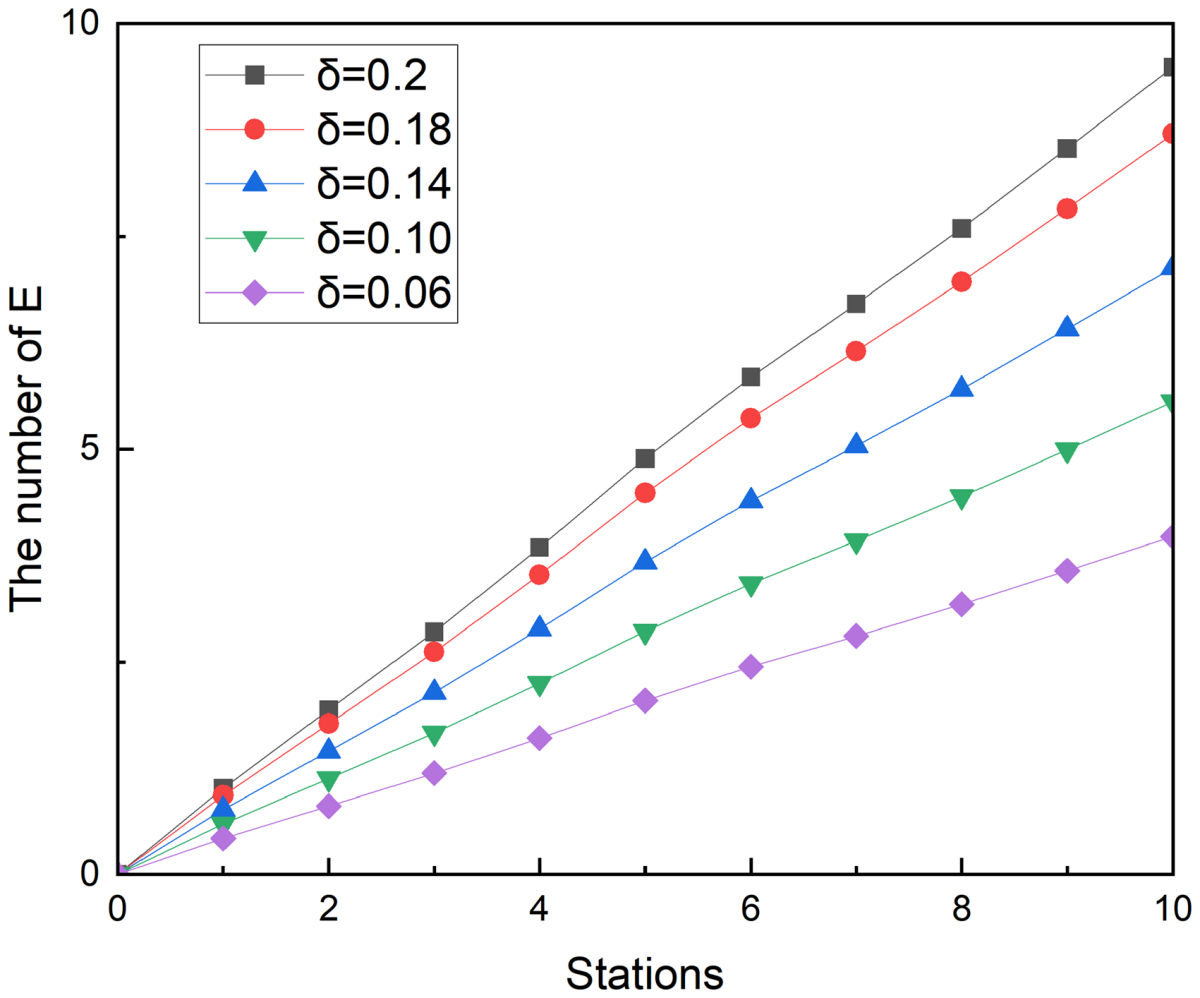
Simulation results of social distancing during off-peak period.

Referring others research, where the passengers move with basic protective measures such as wearing masks, one infected person with Omicron has the ability to infect an average of 3.4 people in a period (32), so the simulated scenario r is set to 3.4. Similarly, because the average infection rate of a close contact event in different population densities is 0.2, $\delta_{1}$ is set to 0.2 (29). At the same time, the infection rate of asymptomatic infected people was gradually reduced by 10, 30, 50, and 70%, that is, $\delta_{2}$ was designed to be 0.18, 0.14, 0.10, and 0.06, respectively. In this way, it is possible to simulate the rate of contagion that decreases as the spacing between the seats of the crowd increases. In order to better observe the effects of various measures on exposed people, a control group was also designed for the simulation, which r was 3.4 when the infected person passed 10 stations, and $\delta_{1}$ and $\delta_{2}$ were both equal to 0.2. This design means that passengers who take the subway will only take basic precautions, such as wearing masks and taking temperatures. Figures 6, 7 show the experimental simulation results of the model for each value during peak period and off-peak period.

Simulating the ridership during the peak hours of the subway is obtained as shown in Figure 6. During the whole operation of the subway, the exposed showed a gradual upward trend. And as the subway passes through the stops, the growth rate of the 1st to 2nd, 3rd to 5th, and 8th to 9th stations is smaller than that of the previous station, and the growth rate of other stations is higher than that of the previous station. Since each curve in the graph has such a feature, it is assumed that this trend feature is not related to the change in $\delta_{2}$ . It was observed that when the number of people getting off at the station was greater than the number of people getting on the subway, the growth rate in that interval decreased. Therefore, it is speculated that the change in the growth rate of the exposed population is related to the change in the density of the people in the subway. With the decreasing of $\delta_{2}$ value, the growth rates of the group of E are controlled. At its peak, the number of probable infections at the last stop dropped from 23.20 to 21.28, 17.44, 13.58, and 9.71, respectively. Compared with the control group, the number of people who could be infected decreased by 8.27, 24.85, 41.48, and 58.14%, respectively. During the off-peak period shown in Figure 7, except for the growth rate of the exposed population at stations 1 to 2 and 4 to 6, the growth rate of other sites is gradually increasing compared with the previous station. Similarly, such changes are also associated with changes in the density of people in subway cars during the off-peak period. The number of probable infections in the last leg dropped from 9.49 to 8.70, 7.13, 5.56, and 3.97, respectively. Compared with the control group, the number of people who could be infected decreased by 8.27, 24.84, 41.46, and 58.13%, respectively.

Compared with the other two scenarios, in general, the implementation effect of social distancing is objective and easy to do. Especially during off-peak hours, when there are fewer passengers in the carriage, it is easier to implement keeping a distance to maintain safety environment, avoid excessive concentration of crowds in poorly ventilated places, and accelerate the spread of diseases. The simulation results show that social distancing has the potential to reduce the infection rate to 58%. The results of the simulation are similar to those of a subway study in the United States, where the effect of crowding on subway infection rates accounts for a major part of the influencing factors (33).

### 5.2 Simulation of the measure of reducing station stops

During the time period studied, the subway in City Z had suspended trains at stations with a large number of infected people during the more severely epidemic period. This measure is known as “reduce the number of stopping station.” That is passing through the station without stopping. And there is no movement of people getting on and off the train. A control group is assumed that the subway is still stopping at 10 stations. The effect of this measure was simulated by observing the changes in the number of people who could be infected by reducing one of nine stopped station during peak and off-peak periods. The detailed simulation experiments are as follows.

**Figure 8:**
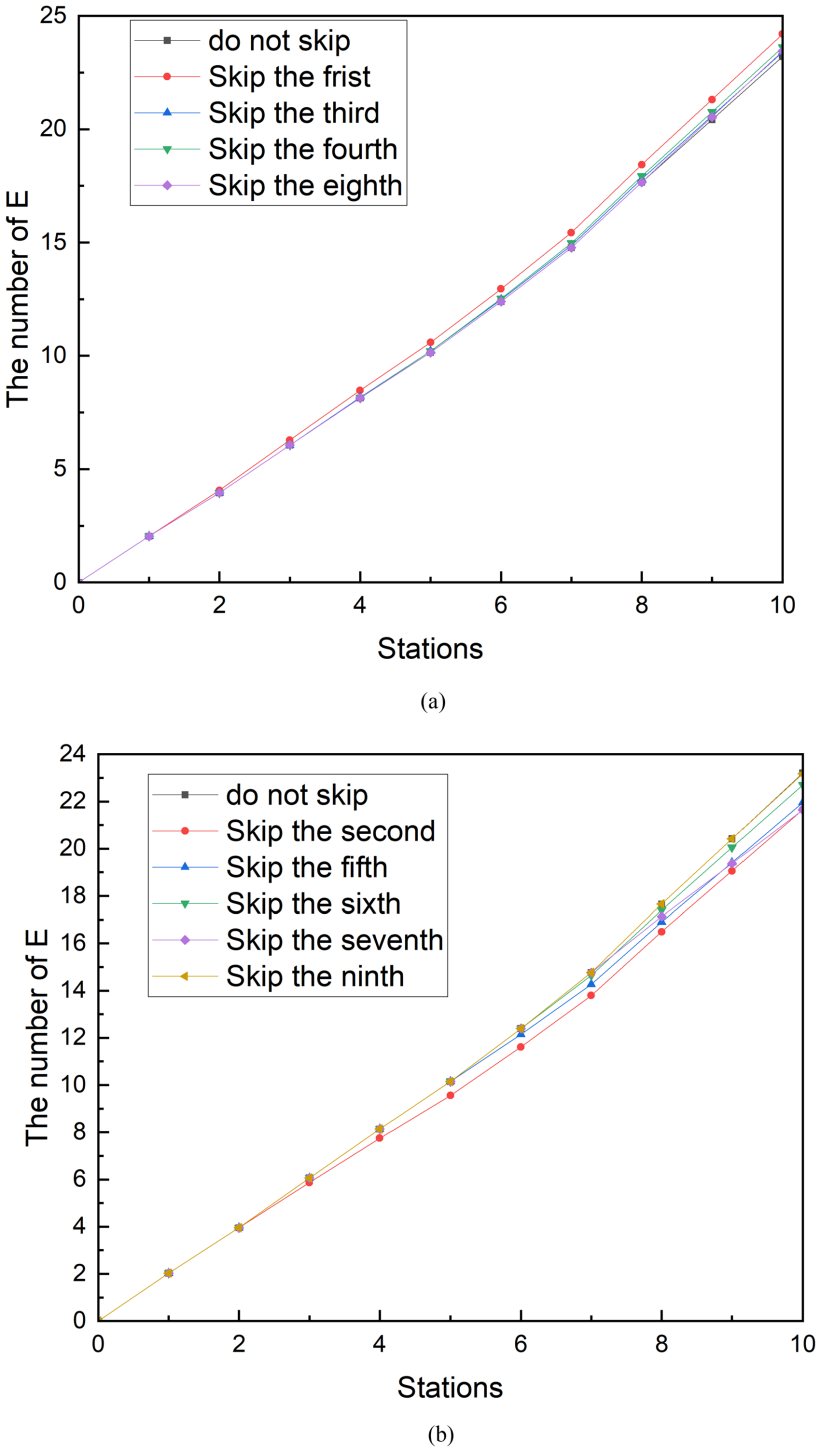
Simulation results of reducing station stops during peak period. **(A)** The number of E is increasing with reducing stopped station. **(B)** The number of E is decreasing with reducing stopped station.

In the simulation scenario of reducing station stops, the curve change of the group E during the peak period and the peak period is not more obvious than the social distancing and does not always reduce the number of exposed people. As shown in Figure 8A, it can be seen that when the subway does not stop at the first, third, fourth, and eighth stations during peak hours, the number of exposed people when the subway reaches the tenth station is higher than the number of exposed people when it does not skip the station. Among them, skipping the first station generated the largest group E, which can reach 24.20 people. Compared with the number of exposed people who did not skip the station, the number of exposed people increased by 4.30%. In contrast, as shown in Figure 8B, when the subway does not stop at the second, fifth, sixth, seventh, and ninth stations, the number of exposed people when the subway travels to the tenth station is less than the number of exposed people which do not skip the station. Compared with the number of exposed people who did not skip stations, the number of exposed people decreased by 6.75%. After observation, it was found that the number of people boarding the subway at the stations where the E group was reduced after being skipped was originally greater than the number of people getting off. Conversely, the number of people getting off at the stations that increased the number of people getting off the subway was greater than the number of people boarding at the stations that caused the increase in group E after being skipped. Therefore, it can be speculated that the effectiveness of the measures to reduce stops is related to the change in the relative density of people in the subway after the implementation. From the reduction rate of 6.75%, it is speculated that the strength of the effect of this relationship is limited. This conclusion is similar to the findings of the other study (33). In the peak period, the seventh station had 257 more people boarding than getting off the bus, which was the most likely to increase the number of people in the subway. Therefore, when the subway does not stop at the seventh station, the increase in the number of exposed people is best controlled.

**Figure 9:**
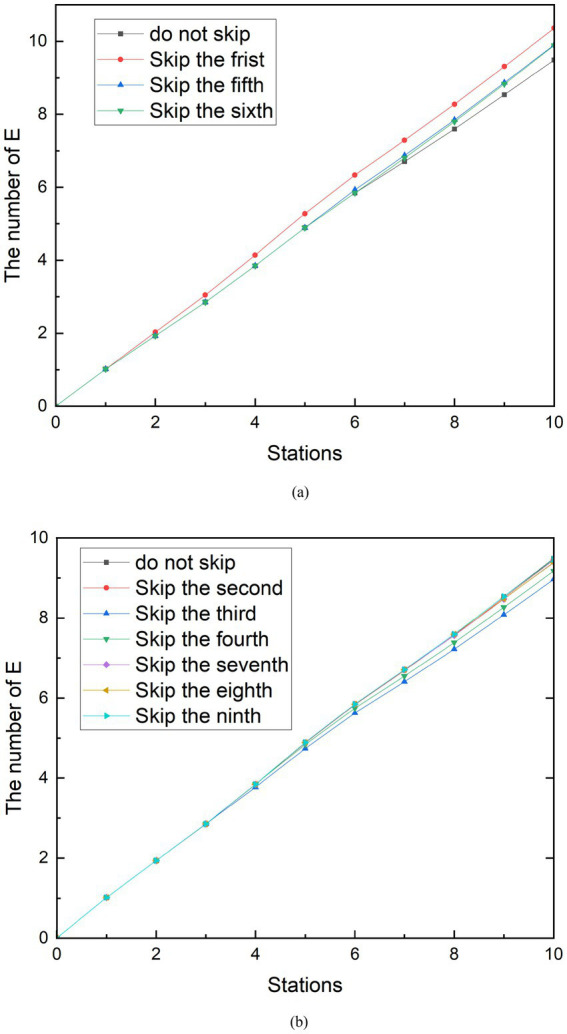
Simulation results of reducing station stops during off-peak period. **(A)** The number of E is increasing with reducing stopped station. **(B)** The number of E is decreasing with reducing stopped station.

The simulation results of measures to reduce stops during off-peak hours can be seen in Figure 9. In addition to the existence of similar results to the peak, another characteristic was found. Similarly, because stopping at stops 1, 5 and 6 reduces the density of people in the subway, skipping these stops increases the number of exposed people. Stops at stops 2, 3, 4, 7, 8, and 9 increase the density of people in the metro car, and skipping these stops reduces the number of exposed people. However, among these sites, the skipped stations that minimized group E was the third, and the number of exposed persons at the tenth station was 8.97, a 5.52% reduction compared to no skipping. The difference in the number of people getting on and off at the third station is 37. Unlike station 6 during peak hours, the third station is not the station that best increases the number of people in the subway. The station that can increase the number of people in the subway during the off-peak period is the ninth station, and the difference in the number of people getting on and off the train is 92. After analysis, the reason for this result is that there is a large difference in the number of stations between the third station and the ninth station, and the subway passes through the third station first. Observing Figure 9, it can be seen that in the case of reducing stops, the curve before the skipping is carried out and the curve without reducing the stops are perfectly fitted. Until the skipping was carried out, the growth rate of the E group suddenly decreased compared to the growth rate of the no skipping group. Therefore, the earlier the shipping is performed, the sooner the growth rate of the E group decreases. Although the difference in the number of people getting on and off at station 9 is greater than that at station 3, the number of exposed people cannot be minimized because the growth rate of group E begins to decrease at the end of the metro journey. A similar phenomenon exists when looking at the simulation of reduced stops during peak periods.

Through the simulation analysis of the measures to reduce stopped stations, it can be found that the implementation effect is affected by the number of people getting on and off the subway at the skipped stations and the order of the skipped stations. Although it can be seen that reducing the number of stopping station play a role on reducing the spread of the epidemic, it cannot play a dominant role. The simulation results demonstrate that reduce stopped stations has the potential to reduce the infection rate to 6.75%.

### 5.3 Simulation of the measure of vaccination

**Figure 10:**
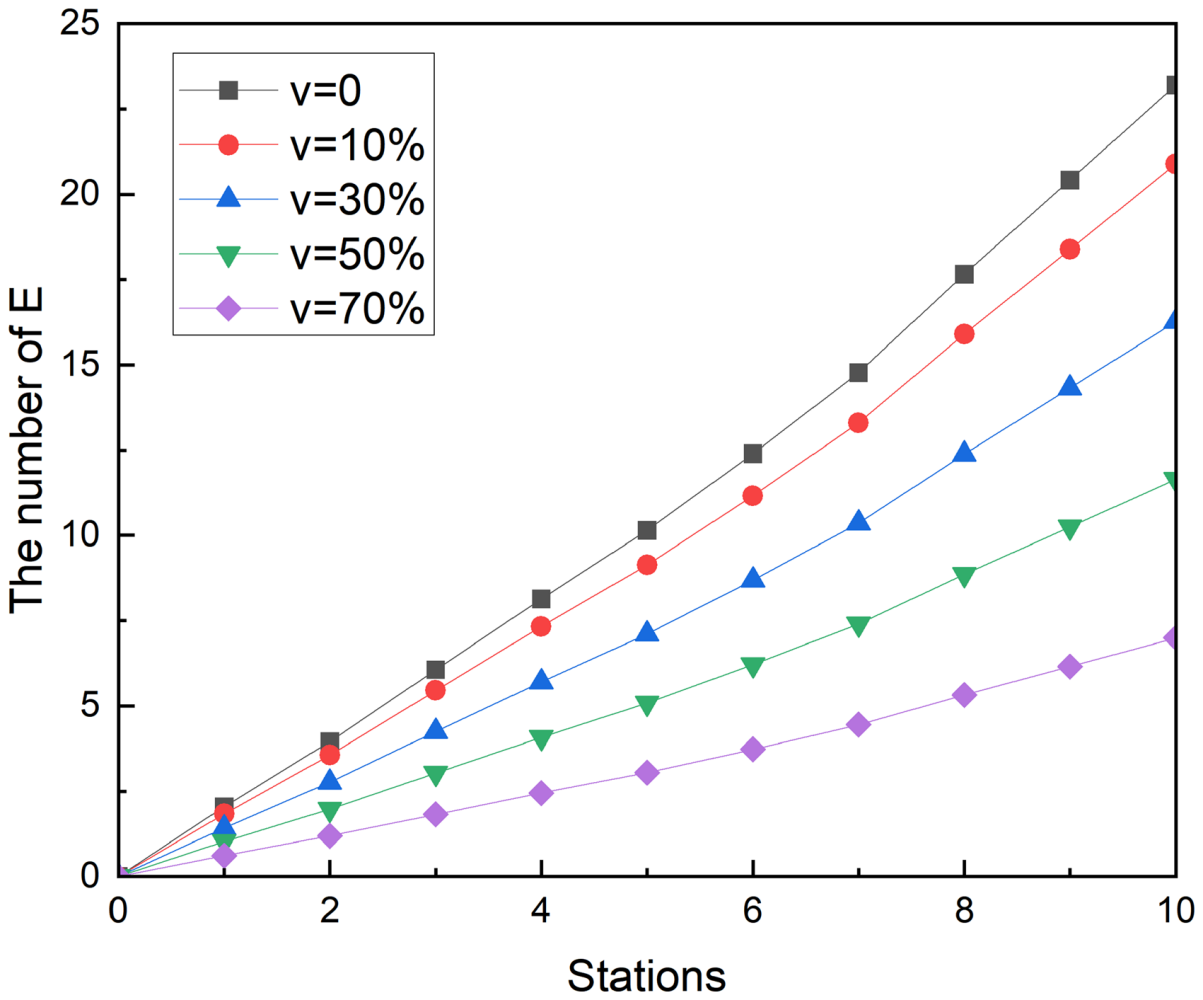
Simulation results of vaccination measures during peak periods.

**Figure 11:**
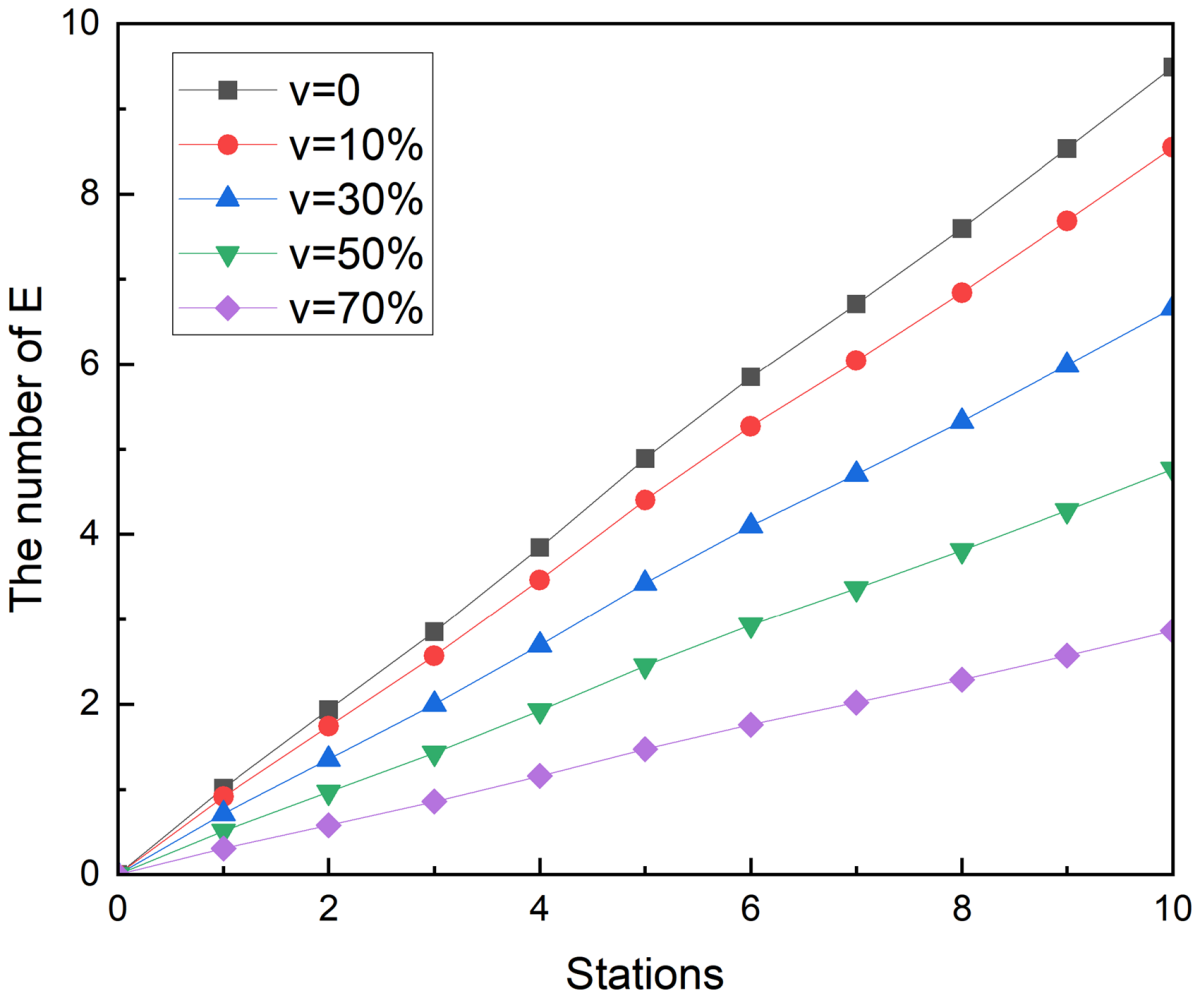
Simulation results of vaccination measures during off-peak periods.

Vaccination is a more cost-effective way to prevent the disease than the lockdown measures taken at the beginning of the pandemic. It starts reducing the spread of COVID-19 from the infection channel of the susceptible person, establishes the body’s epidemic prevention barrier, protects the susceptible population and reduces the risk of infection. Since 2021, Chinese government has been calling for susceptible people to get vaccinated, and the number of doses has been developed from one dose to the current four. Studies have shown that vaccines can protect 70% of people and reduce the risk of infection (34). To get a picture of the impact of vaccination on the spread of COVID-19 in the metro, a new parameter was added to the study, vaccine coverage v. The hypothesis is that when a susceptible population with a proportion of V is vaccinated, the proportion of susceptible people who can be converted into exposed people is reduced. With this method, we can see the results of the vaccine in the simulation experiments in Figures 10, 11. Likewise, r is assumed to be 3.4. $\delta_{1}$ and $\delta_{2}$ are assumed to be 0.2. Vaccine coverage v was set at 10, 30, 50, and 70%, respectively.

According to the simulation results, it can be found that the implementation effect of vaccination measures is obvious. With the increase in vaccine coverage, the increase in the number of exposed people has slowed down significantly. In the end, the number of possible infections dropped from 23.20 to 20.90, 16.28, 11.65, and 7.00, respectively. The number of people who could be infected decreased by 9.93, 29.84, 49.81, and 69.84%, respectively, compared with the control group. Similar to other measures, changes in the growth rate of exposed groups correlated with changes in the density of people in subway cars. Because the number of people at the next station is higher than the number of people at the previous station, the growth rate of stations 1 to 2, 3 to 5, and 8 to 9 is smaller than that of the previous station.

The trend of simulation in off-peak is not significantly different from the scenario simulation during peak hours. In the end, the number of possible infections dropped from 9.49 to 8.55, 6.66, 4.77, and 2.86, respectively. Compared with the control group, the number of people who could be infected decreased by 9.92, 29.82, 49.79, and 69.82%, respectively. The growth rate of the exposed population also increased with the increasing density of the people in the subway car. The simulation results show that vaccination has the potential to reduce the infection rate to 69.84%.

## 6 Discussion

### 6.1 Comparative analysis of different measures

The purpose of studying the spread of COVID-19 in the subway is to identify the key factors in the transmission process of the epidemic, and then different measures can be taken to avoid infection according to different key factors, by exploring the transmission mechanism. For example, keeping a distance is adopted to reduce the probability of susceptible people infected after connecting with the infection. For the restrictions on the infected, nucleic acid testing and isolation of infected people are commonly used. In the early stage of the epidemic, such measures can still be taken to suppress the spread of the disease, but with the mutation of the virus itself, the characteristics are gradually changing, and the economic cost of stricter lockdown measures is higher, so the epidemic prevention policy has now been changed to keep health without affecting people’s normal work and life. Vaccination, on the other hand, is a more economical measure that has less impact on people’s lives, and it directly defends against the COVID-19 virus by protecting susceptible people. However, the specific implementation of these protective measures will take a period of practice to see the results, but through the establishment of mathematical models, the key factors affecting the epidemic can also be identified, the effectiveness of the current epidemic prevention measures can be analyzed, and the decision-making basis for the government and the subway can be provided.

**Figure 12:**
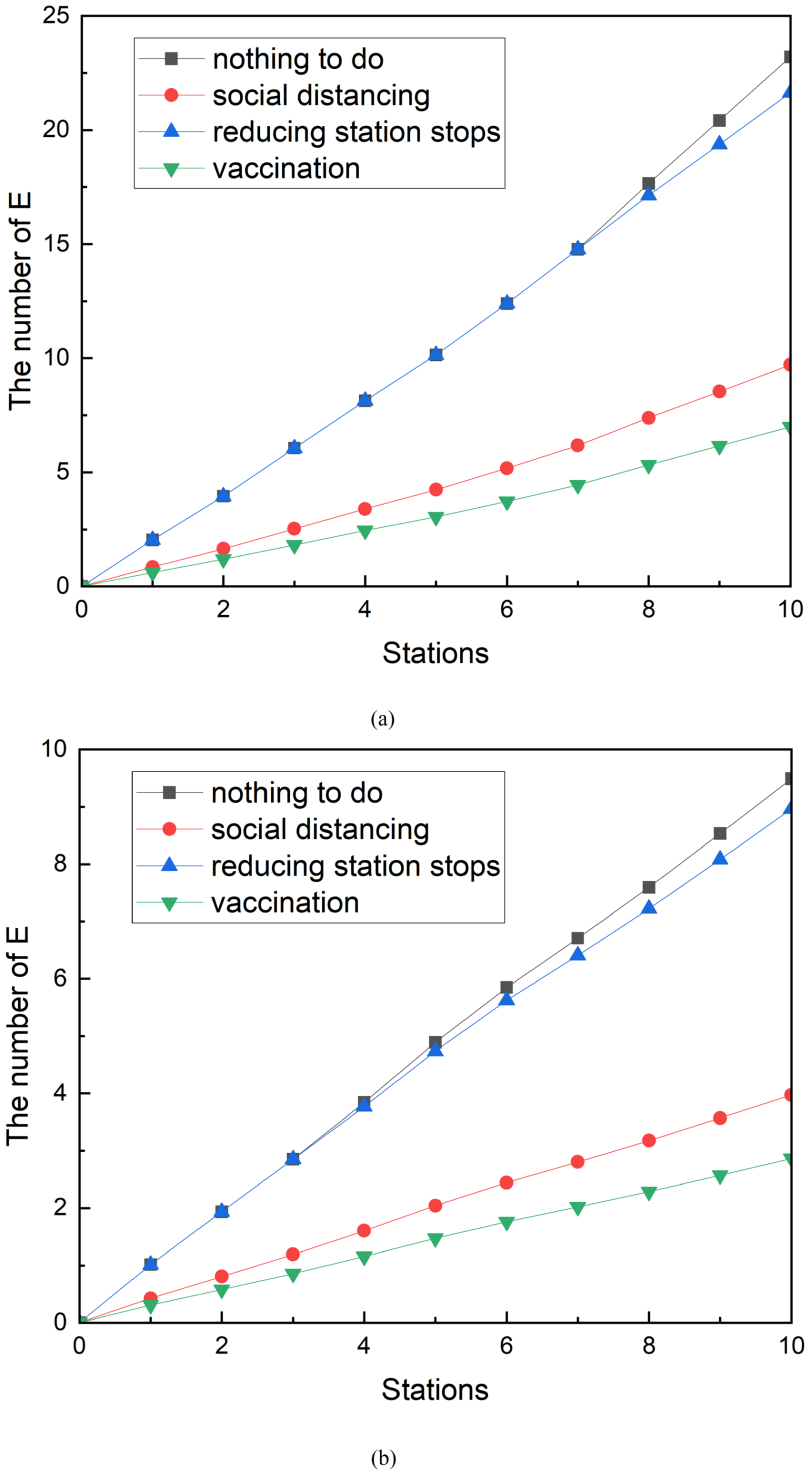
Comparison of the implementation effects of various measures. **(A)** Peak period. **(B)** Off-peak period.

The results of Figure 12 can be obtained by comparing the effects of the implementation of protective measures such as keeping a distance, reducing the number of stopping station, and vaccination. In the measure comparison, when simulating the social distancing, $\delta_{2}$ is equal to 0.06. Vaccine coverage V is equal to 70%. During peak hours, the reducing stopped stations measures are specifically designed to skip the seventh stop, which minimizes the number of E-groups after the stops are skipped. During off-peak hours, the third stop is designed to be skipped, which also minimizes the number of exposed groups in this scenario.

At the peak, the curve of the vaccination measures was the slowest to grow between the curve of the different measures. Until the train reaches the 10th station, the entire curve has no intersection with other curves. This indicates that the implementation of vaccination is the most effective between three measures. It can effectively reduce the number of group E, and achieve the effect of epidemic prevention. In addition, the simulation results of social distancing measures are second only to vaccination. It can also effectively reduce the number of exposed people. The E-curve that reduces stops turns at the sixth station and ends up being smaller than the E-curve where no additional measures are taken. But the trend is not as obvious as the other two curves, which indicates that although the reducing stopped stations have played a certain role, the effect is not good compared with other measures. The final number of group E can be seen at station 10, 9.71 exposed people with social distancing, 7.00 exposed people with vaccination measures, and 21.64 with reducing stopped stations. The exposed people decreased by 13.49, 1.57, and 16.20, respectively, compared to those with only basic protection measures. The group E with epidemic prevention measures decreased by 58.14, 6.75, and 69.83%, respectively, compared with those who only took basic protection measures.

Among the curves of E under different measures during the off-peak period, the E curve of vaccination was still the lowest average growth rate among all curves. In addition, the change in the E-curve of the operation measures of reducing the number of stopping station is similar to that of the peak period, and the trend is similar to that of the curve of the number of potential infected people who have taken only basic measures, and the number of people is also relatively close. This indicates that the reducing the number of stopping station measures are relatively poorly implemented in the measures studied. In terms of the final number of probable infected person E, at station 10, the number of exposed people with keeping a distance was 7.88, the final number of exposed people with vaccination measures was 8.00, and the number of people with reducing the number of stopping station was 14.52. Compared to the number of possible infections with only basic protection measures, the number of group E decreased by 7.17, 7.05, and 0.53, respectively. The number of exposed people with epidemic prevention measures decreased by 47.64, 46.84, and 3.52%, respectively, compared with those who only took basic protection measures.

The implementation of keep a distance is the best measure found by contrasting vary the number of exposed people under different measures. Vaccination measures can also work well, reducing the number of people likely to be infected in the subway by about 20 to 40 percent. In addition, the advantage of vaccination measures is that they can also be used in scenarios other than the subway for a period of time after vaccination. Compared with social distancing and vaccination, the implementation effect of reducing the number of stopping station is difficult to meet the demand, but it can also have some effect. Especially during the epidemic period, the implementation of reducing the number of stopping station in areas with a large number of infected people has helped to reduce the number of infected people to take public transportation to reduce the risk of infection.

### 6.2 Comparative analysis of different combination measures

**Figure 13:**
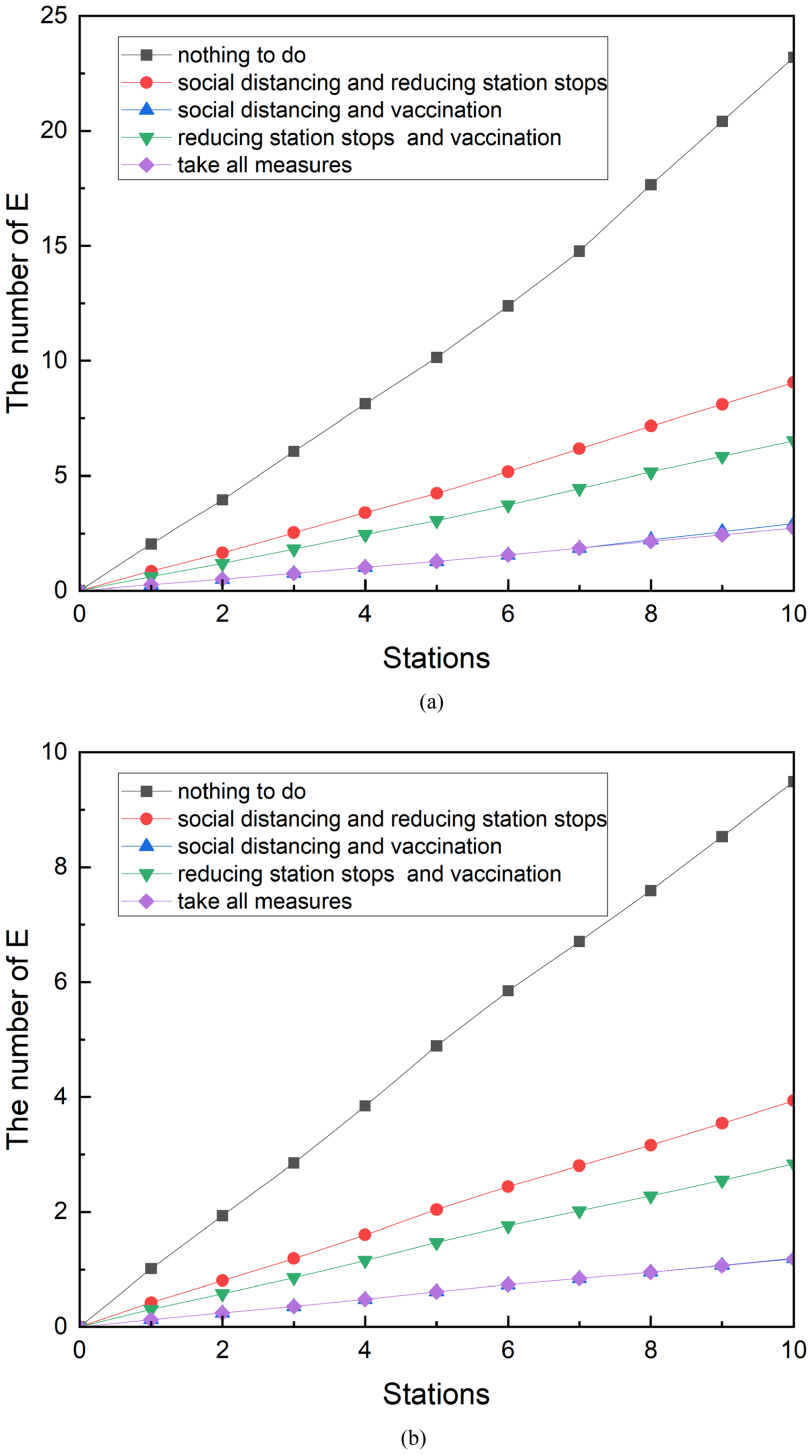
Comparison of the implementation effects of combination measures. **(A)** Peak period. **(B)** Off-peak period.

In practice, it is not common for authorities to implement a single measure to control the COVID-19 pandemic. The subway management department of Z city has simultaneously implemented measures such as QR code management, social distancing, supervising passengers to wear masks, and reducing stops to control the development of the virus after the resumption of production. Vaccination is one of the measures that the Chinese government has used to contain the spread of the epidemic throughout the country. Therefore, the epidemic prevention effect of the combination of simulated measures is also an effective way to manage the epidemic control. A control group was designed to compare the effects of the combined measures. The scenarios of the combination of social distancing and reduced stops, the combined implementation of social distancing and vaccination, the combined implementation of reduced stops and vaccination, and the combination of three measures were set up during peak periods. Similarly, the same is done during the off-peak phase of subway traffic. The results of the comparison are shown in Figure 13.

Although the data volume of the final E-population is different, the effects of various combinations of measures are consistent between the peak and off-peak periods. During peak periods, 9.06 people were exposed after the implementation of the combination of social distancing and reduced stops. The number of people exposed from the combination of reduced stops and vaccination was 6.53. The number of people exposed after the implementation of social distancing and vaccination was 2.92. The number of people exposed after the combination of reduced stops and vaccination was 6.53. The number of exposed persons after the combined implementation of the three measures was 2.72. The combination of measures was compared with the control group, and each of them was reduced by 60.96, 87.41, 71.86, and 88.26%. The simulation results can be found to have the lowest number of exposed people when the three methods are combined. However, there is only a 0.2 gap between the results of the combined social distancing and vaccination measures. Compared with the combined measures of social distancing and vaccination, the combined implementation of the three measures only reduced by an additional 0.84%.

The implementation of various joint measures during the off-peak period is similar. The number of exposed persons from the implementation of the combination of social distancing and reduced stops was 3.94. The number of exposed people resulting from the joint implementation of social distancing and vaccination is 1.20. The number of exposed people after the implementation of the combination of reduced stops and vaccination was 2.84. The number of exposed persons after the combined implementation of the three measures was 1.18. The combined measures were compared with the control group and were reduced by 58.54%, 87.41%, 70.11, and 87.53%. Compared with the combined measures of social distancing and vaccination, the combined implementation of the three measures only reduced the effect by an additional 0.12%.

When combined with individual measures and combined measures, it can be found that four of three the combined measures generate fewer E populations than the individual implementation measures. Only when vaccination is carried out independently will produce fewer exposed people than the number of exposed people produced by social distancing combined with reduced stops. Based on the simulation model established in this study, the number of exposed groups was observed after 10 stations of subway operation. With the goal of reducing the number of exposed groups, all measures were ranked from obvious to ineffective: taking all measures, social distancing and vaccination, reducing stops and vaccination, vaccination, social distancing and reducing stops, social distancing, and reducing stops.

### 6.3 Suggested measures

In this study, we discussed the effectiveness of relevant protective measures in preventing and controlling the spread of COVID-19 by establishing a model of COVID-19 transmission in the SEIA subway and setting up different scenarios to study the key factors of COVID-19 transmission. Provide reasonable protective measures and suggestions for the prevention and control plan of public health emergencies, so as to achieve the purpose of better serving the prevention and control decision-making of the research results. As one of the main parts of urban public transportation, the subway is an important guarantee for the normal operation of society. The subway management department shall assume the social responsibility of epidemic prevention while ensuring the normal travel of passengers. Combined with the scenario simulation analysis in this chapter, the following three suggestions can be put forward for the protection measures to be considered by subway operation and personal travel:

Announcements in the station urge passengers to keep a distance and to take ventilation and disinfection measures before and after train operation.

Announcements in subway stations and carriages play warm reminders on a loop, urging passengers to sit at intervals. Passengers are reminded to take basic precautions. Travelers are required to wear masks on the subway. Safety inspectors can be placed in waiting areas and in the carriages, and passengers are instructed to wear masks, not to take them off at will, and to sit as far apart as possible. Strengthen various health and epidemic prevention measures, and regularly disinfect areas with large gatherings of people and public areas that are prone to the spread of the epidemic. Trains are forced to be fully disinfected and ventilated before operation. Especially during the epidemic period, it is possible to consider extending the ventilation time or strengthening the disinfection and cleaning of key areas.

Strengthen the protection of key hub stations, provide public epidemic prevention materials in the stations, and enhance the protection awareness of staff.

For some large transfer stations or key stations with a large passenger flow, monitoring should be strengthened. Especially during the epidemic period, the subway can adopt operational adjustment measures such as “reducing stop stations” or “limiting passengers flow” to reduce the risk of disease transmission. At the same time, key stations need to be disinfected. The subway in the station can provide public anti-epidemic materials for passengers to use, such as placing hand sanitizer and adding mask collection machines. In addition, subway workers, as a group with a greater risk of exposure, are also an important part of the epidemic prevention process. The subway management department shall regularly organize employees to conduct epidemic prevention and safety awareness training, so as to strengthen employees’ ability to deal with public health emergencies and ensure the safe and orderly operation of trains during the epidemic period.

Increase vaccination rates, stagger subway rides, and maintain social distancing.

In order to prevent the epidemic, citizens should respond to the call of the state and get vaccinated in time. In particular, older adults and children, two susceptible groups, should be vaccinated in time to establish their own epidemic prevention protection. As ordinary citizens, they should understand the relevant knowledge of epidemic prevention and control, and look at the spread of diseases with a scientific and objective attitude. When traveling by subway, if there is no rigid travel demand, you can consider off-peak travel to avoid peak passenger flow periods. If you need to take the subway during heavy traffic periods, you should try to maintain a safe distance. For short rides, consider standing to reduce contact with high-use public facilities such as seats.

## 7 Conclusion

Based on the SEIA model that simulates the spread of COVID-19 in the subway, this paper identifies the key factors in the transmission, and simulates the data source obtained from the subway passenger flow in Z city to explore the effectiveness of the protective measures related to subway travel. In the survey data of the subway passenger flow in Z city, because the subway passengers mainly take the subway for commuting needs during the normal epidemic prevention period, although some people have arranged off-peak commuting, it also shows the regular peak and off-peak phenomenon. On the basis of the classical SEIR model, the SEIA model divides the infected people into symptomatic infected people and asymptomatic infected people, which mimics the infected people of COVID-19. At the same time, for the characteristics of the subway crowd movement, the rehabilitation crowd was abandoned and the change of the crowd getting on and off the train was increased. The number of people getting on and off the train is randomly generated based on the survey data of the subway in Z city. The proportion of various types of people in the boarding population is approximately replaced by the proportion of the number of confirmed cases in the resident population in city Z. However, there is still a difference between the proportion of the number of confirmed cases in the resident population in city Z and the proportion of susceptible people, infected people, and asymptomatic infections in the subway commuting demand group. This gap may have an impact on the outcome. Therefore, a more accurate estimate of the number of various types of people who need to take the subway is the direction that can be further studied and accurately simulated results.

This model can be used as a more general model to evaluate common subway epidemic prevention measures. The simulation results show that the implementation of different protective measures, such as social distancing, reducing stopped station and vaccination, can affect the number of people who may be infected. According to the results of simulation analysis, Peak and off-peak subway ridership can affect the number of exposed people. However, the trend of the exposed population is similar to the growth rate of the exposed population after the implementation of epidemic prevention measures in the two passengers flow scenarios. It can be seen that the vaccination is the most effective protective measure in the effect of the implementation of individual countermeasures, which can reduce the number of exposed people by up to about 70%. Social distancing is second to vaccination, and it is also significant. During subway operation, social distancing measures can reduce the exposure of people by about 58% after 10 subway stops. In contrast, the reducing stopped stations do not significantly reduce the number of people who may be exposed, but they also have a reduction effect on the potential infections. Among the combined measures, the number of exposed persons was the least after the combined implementation of the three means, which can reduce the number of exposed people by up to about 88%. However, compared with the combined measures of social distancing and vaccination, the combined implementation of the three measures only reduced by an additional less than 1%. The study has contributed to the formulation of epidemic prevention measures in subway operation, and also provided a reference for other major public health emergency response measures.
